# Trifecta Outcomes of Robot-Assisted Partial Nephrectomy Using the New Hugo™ RAS System Versus Laparoscopic Partial Nephrectomy

**DOI:** 10.3390/jcm13072138

**Published:** 2024-04-07

**Authors:** Francesco Prata, Alberto Ragusa, Francesco Tedesco, Matteo Pira, Andrea Iannuzzi, Marco Fantozzi, Angelo Civitella, Roberto Mario Scarpa, Rocco Papalia

**Affiliations:** Department of Urology, Fondazione Policlinico Universitario Campus Bio-Medico, 00128 Rome, Italy; alberto.ragusa@unicampus.it (A.R.); francesco.tedesco@unicampus.it (F.T.); matteo.pira@unicampus.it (M.P.); andrea.iannuzzi@unicampus.it (A.I.); marco.fantozzi@unicampus.it (M.F.); a.civitella@policlinicocampus.it (A.C.); r.scarpa@policlinicocampus.it (R.M.S.); rocco.papalia@policlinicocampus.it (R.P.)

**Keywords:** laparoscopic surgery, robotic surgery, partial nephrectomy, trifecta, Hugo RAS

## Abstract

**(1) Background**: Laparoscopic partial nephrectomy (LPN) is still performed in many referred urological institutions, representing a valid alternative to robot-assisted partial nephrectomy (RAPN). We aimed to compare trifecta outcomes of LPN and RAPN with the Hugo™ RAS System. **(2) Methods**: Between October 2022 and September 2023, eighty-nine patients underwent minimally invasive partial nephrectomy (group A, RAPN = 27; group B, Laparoscopic PN = 62) for localized renal tumors at our Institution. Continuous variables were presented as median and IQR and compared by means of the Mann–Whitney U test, while categorical variables were presented as frequencies (%) and compared by means of the χ^2^ test. **(3) Results**: Group A showed a higher rate of male patients (81.5% vs. 59.7%, *p* = 0.04) and a higher trend towards larger clinical tumor size (34 vs. 29 mm, *p* = 0.14). All the other baseline variables were comparable between the two groups (all *p* > 0.05). Regarding post-operative data, group A displayed a lower operative time (92 vs. 149.5 min, *p* = 0.005) and a shorter hospital stay (3 vs. 5, *p* = 0.002). A higher rate of malignant pathology was evidenced in group A (77.8% vs. 58.1%, *p* = 0.07) as well as a lower trend towards positive surgical margins (3.7% vs. 4.8%, *p* = 0.82), even if not statistically significant. **(4) Conclusions**: The rate of trifecta achievement was 92.6% and 82.3% for group A and B (*p* = 0.10), respectively. In terms of trifecta outcomes, RAPN using the Hugo™ RAS System showed comparable results to LPN performed by the same experienced surgeon.

## 1. Introduction

Renal Cell Carcinoma (RCC) is the most common type of kidney cancer, with an increasing incidence globally, representing about 3% of all cancers in Western countries, with the most prevalent portion composed of small renal masses due to early diagnosis or incidental discovery during other exams [1,2,3,4,5]. Although radical nephrectomy has represented the gold standard for the treatment of RCC for years, the advent of nephron-sparing surgery with its expanded indications has consistently reduced the number of renal masses treated with a radical intent. Thus, partial nephrectomy results make it the preferred technique whenever feasible [6,7,8].

In the field of minimally invasive techniques, laparoscopic and robotic surgeries represent the most favored approaches. Laparoscopic partial nephrectomy (LPN) emerged as a mainstay, offering patients a less invasive option while ensuring effective oncological outcomes [9,10,11]. However, the steep learning curve and technical difficulties associated with laparoscopy make it intricate and not always easily reproducible. For this reason, robot-assisted partial nephrectomy (RAPN) has taken the upper hand in the field of minimally invasive nephron-sparing surgery (NSS), providing benefits for both patients and surgeons. RAPN empowered surgeons to overcome the technical challenges associated with laparoscopy, becoming a widely recognized standard option, pushing its indications to all clinical T1 RCCs and, in specific cases, even for T2 renal masses [12,13,14,15,16,17].

In this scenario, since DaVinci platform patent has expired breaking the robotic market monopoly, more robotic systems have emerged offering surgeons supplementary surgical opportunities [18]. The Hugo™ RAS System by Medtronic is one of the most recent platforms, demonstrating interesting results across several procedures, such as RAPN [19]. Nevertheless, consistent and reproducible data regarding NSS are still lacking.

Based on this background, we aimed to report our experience in a large minimally invasive partial nephrectomy series including either laparoscopic and robotic approaches, focusing on the transition from the first to the latter and comparing the achievement of a novel trifecta between LPN and RAPN.

## 2. Materials and Methods

### 2.1. Study Design and Patient Population

Between October 2022 and September 2023, eighty-nine patients underwent minimally invasive partial nephrectomy (PN) at our Institution. This cohort was divided into two groups: group A (RAPN = 27) and group B (LPN = 62). All consecutive patients eligible for minimally invasive partial nephrectomy were included in the study. The only exclusion criteria were gross hematuria or tumor infiltration observed on conventional imaging, rendering the patient ineligible for a nephron-sparing surgery. After obtaining informed written consent before the procedure, comprehensive baseline, pre-, intra-, and post-operative data were meticulously collected. Both laparoscopic and robotic procedures were performed by a single experienced surgeon in minimally invasive surgery. Regarding robotic assistance with the new platform, the surgeon, along with bed assistants, scrub nurses, and the entire surgical team involved in the procedure, received comprehensive technical training on the Hugo™ RAS System. This training was provided by Medtronic at the ORSI Academy in Aalst, Belgium.

### 2.2. Endpoints, Outcome, and Statistical Analysis

The primary objective of the study was to compare trifecta outcomes of off-clamp LPN vs. off-clamp RAPN using the Hugo™ RAS System. The secondary aim was to assess the feasibility and safety of this novel platform during the transition of a single surgeon, who had no prior robotic experience, from laparoscopy to robot-assisted surgery. The study utilized the trifecta as a comprehensive measure, defined by the coexistence of negative surgical margin status, no Clavien–Dindo grade ≥3 complications, and eGFR decline ≤30% [20]. The body mass index (BMI) was calculated as weight in kilograms divided by height in meters, squared (kg/m^2^), and post-operative complications were reported according to the Clavien–Dindo classification [21]. The estimation of glomerular filtration rate (eGFR) was calculated using the CKD-EPI formula. Perioperative data, including operative, console, and docking time; estimated blood loss; and post-operative complications, were recorded. Continuous data were presented as median and interquartile ranges (IQR), while frequencies and proportions were used for categorical variables. Continuous and discrete variables were compared using the Mann–Whitney U test and the Chi-square test, respectively. A two-sided *p*-value < 0.05 was considered statistically significant. STATA (StataCorp. 2021. Stata Statistical Software: Release 17. College Station, TX, USA: StataCorp LLC) was used for statistical analyses.

### 2.3. Trocar Configuration and Surgical Procedure

Following general anesthesia and trans-urethral bladder catheter placement, patients were positioned in a lateral decubitus manner, adopting a modified extended flank position with a slight flexion (45°). All procedures were conducted by a single highly experienced surgeon.

Trocar configuration in LPN consisted of a main 12 mm laparoscopic trocar for the endoscope on the pararectal line lateral to the umbilicus or slightly far depending to the renal mass position, which provides a central and stable vantage point. Auxiliary trocars, ranging from 5 to 12 mm, were then strategically positioned, often forming a triangular or quadrangular arrangement around the primary trocar. This geometric set-up facilitates optimal instrument triangulation, minimizing clashing and interference during the procedure. 

**Figure 1:**
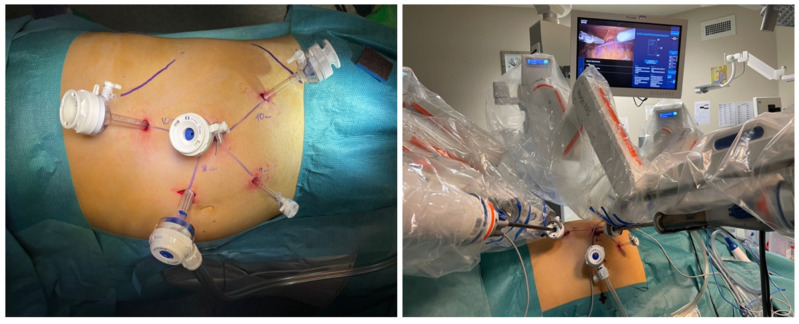
Surgical robotic set-up: trocar position and arm carts triangulation.

Trocar configuration in RAPN using the Hugo™ RAS System was recently described in a three-arms setting [14,22], which involved an initial 11 mm robotic camera trocar along the pararectal line, positioned approximately 14 ± 2 cm away from the umbilicus. Two additional 8 mm operative robotic ports were placed under direct vision caudal to the endoscope trocar, which ensured appropriate distances from the optical port. Two 12 mm laparoscopic ports for the bed assistant were carefully situated between the robotic ports, maintaining a minimum separation distance of 8 cm. A safety margin of 2 cm from all bony prominences was observed (Figure 1). A standard intra-abdominal pressure of 12 mmHg was achieved through pneumoperitoneum induction.

For both the LPN and RAPN procedures, the key steps included incision along the Toldt line, complete kidney mobilization, and a straightforward approach to the renal mass. An off-clamp technique was employed in every case, and bleeding from the resection site was managed using LigaSure™ Atlas for LPN and monopolar scissors for RAPN. Renorraphy was performed with a 2/0 Monocryl single-running suture, employing a sliding-clip technique. Hemostasis was achieved with hemostatic agents if needed. After successful normotensive control, the closure of Gerota’s fascia was executed and a single drainage tube was inserted. 

### 2.4. Post-Operative Management and Follow-Up

Abdominal drainage and trans-urethral catheter were removed between the 2nd and 3rd post-operative day before discharge. According to EAU guidelines, follow-up includes routine blood tests, renal function monitoring, CT scans at 6-month intervals for the first two years, and vigilant surveillance for complications. After the first two years, CT scan was conducted yearly. Regular assessments, particularly within the first few years, aid in detecting potential issues and improving long-term survival [6].

## 3. Results

All patients successfully underwent minimally invasive PN without the need for conversion or additional port placement. Baseline and demographic data, as presented in Table 1, revealed for group A a higher rate of male patients (81.5% vs. 59.7%, *p* = 0.04) and a trend towards larger clinical tumor size (34 vs. 29 mm, *p* = 0.14). 

Overall, baseline variables between the two groups were comparable (*p* > 0.05). Regarding post-operative outcomes (Table 2), group A demonstrated favorable results in terms of operative time (92 vs. 149.5 min, *p* = 0.005) and a significantly shorter hospital stay (3 vs. 5 days, *p* = 0.002).

Estimated blood loss was comparable between the two groups (*p* = 0.20), as well as perioperative complications (*p* = 0.07). Although group A showed a higher rate of malignant pathology (77.8% vs. 58.1%, *p* = 0.07) and a lower trend towards positive surgical margins (3.7% vs. 4.8%, *p* = 0.82), these differences were not statistically significant. No significant differences were reported between pre- and post-operative renal function deterioration (*p* = 0.85). Hemoglobin levels at discharge were slightly higher in the LPN group even if not statistically significant (*p* = 0.09). Creatinine and eGFR at discharge (*p* = 0.67, 0.85; respectively), pathological size of tumors (*p* = 0.23), and the rates of benign and malignant pathology (*p* = 0.07), as well as distribution across histological subtypes (*p* = 0.27), demonstrated no statistically significant differences between the groups. While positive margins were infrequently encountered overall (4.5%), the pT stage distribution revealed no significant differences between the groups (*p* = 0.11). At the last follow-up, both creatinine and eGFR displayed similar values (*p* = 0.53 and 0.97, respectively), with a trifecta achievement rate of 85.4% for the entire cohort. When splitting for surgical technique, the trifecta achievement rate was 92.6% for group A and 82.3% for group B (*p* = 0.10).

## 4. Discussion

In the era of NSS, PN is considered the treatment of choice for T1 RCC. This approach, by preserving kidney function more effectively in the long term, has the potential to limit the incidence of cardiovascular disorders, the development of end-stage renal disease (ESRD), and consequently, the need for hemodialysis [23,24,25,26,27,28].

Concerning T2 renal masses, while indications for minimally invasive treatment are expanding, EAU guidelines recommend PN in large renal masses for specific situations. This is particularly crucial for patients with a solitary kidney, bilateral renal tumors, or chronic kidney disease (CKD) where sufficient parenchymal volume needs to be preserved to avoid post-operative renal function deterioration. Recent studies have further supported the satisfactory outcomes of treating T2 renal masses with PN whenever technically feasible [6,16,29,30].

In a retrospective propensity-score-matched study, Chang K.D. et al. compared open, laparoscopic, and RAPN among 1308 patients. Following a median 5-year follow-up, results revealed similar oncological outcomes across the approaches. Comparable rates of local recurrence (*p* = 0.882), distant metastasis (*p* = 0.816), and cancer-related deaths (*p* = 0.779) were observed. When analyzing perioperative outcomes, RAPN demonstrated superiority over open partial nephrectomy and LPN, showing lower estimated blood loss (EBL, *p* = 0.040 vs. 0.025; respectively) and a shorter hospital stay (*p* = 0.008). Additionally, a significantly lower incidence of CKD upstaging compared with LPN (20.55% vs. 32%; *p* = 0.035) and open approach (20.5% vs. 33.6%; *p* = 0.038) was observed. The 5-year CKD-free survival rate was significantly higher in the RAPN group (78.4%) compared with the LPN (58.8%) and open partial nephrectomy (65.8%) groups (log-rank *p* = 0.03) [31].

However, the absolute superiority of RAPN over LPN did not appear to be confirmed, especially when performed by highly experienced surgeons, as demonstrated by Alimi Q. et al. In their prospective multicentric study, perioperative, short-term oncological, and functional outcomes appeared to be broadly comparable [32]. In fact, transfusion rates were similar between the two groups (8% vs. 6%; *p* = 0.33). Moreover, positive surgical margin rates were comparable in the RAPN and LPN groups (2% vs. 6%; *p* = 0.36). After a median follow-up of 19 and 14 months in the RAPN and LPN groups, respectively (*p* = 0.38), recurrence-free survivals did not differ significantly (*p* = 0.94) [32].

Despite these results, Choi J.E. et al. in their systematic review showed a significantly lower rate of conversion to open (*p* = 0.02) and radical surgery (*p* = 0.0006), shorter warm ischemia time (*p* = 0.005), a smaller change in estimated GFR after surgery (*p* = 0.03), and a shorter length of hospital stay (*p* = 0.004) in the robotic group. Moreover, in this meta-analysis of 23 studies encompassing 2240 patients, there was no significant difference between the two groups in the complications of Clavien–Dindo classification grades 1–2 (*p* = 0.62), Clavien–Dindo classification grades 3–5 (*p* = 0.78), change of serum creatinine (*p* = 0.65), operative time (*p* = 0.35), EBL (*p* = 0.76), and positive margins (*p* = 0.75) [33]. However, despite the wide spread of robot-assisted surgery in the NSS field, traditional LPN is still performed in many referred urological institutions representing a valid alternative for treating renal tumors. 

While the laparoscopic approach is still preferred mainly due to lower costs compared to robotic surgery, the advent of new platforms is changing the landscape of the medical market. The Hugo™ RAS System by Medtronic was recently introduced as a novel robotic tool characterized by four independent arm-cart dockings and augmented modularity, showing promising initial results [13,19,22]. The system consists of a console with two arm controllers operated with a grip similar to a pistol; a footswitch controlling the camera, energy source, and reserve arm; and four separate arm carts with six joints each for an increased range of motion. It utilizes a specific pair of 3D glasses for head-tracking technology. Despite the rapidly growing literature, in which results are mostly focused on radical prostatectomy, homogeneous and robust data from a large series of RAPN using Hugo™ RAS are still lacking.

Our manuscript aims to enhance current scientific knowledge on robotic surgery in partial nephrectomy, specifically by utilizing the novel platform provided by Medtronic. In our setting, the same experienced surgeon performed both RAPN and LPN through an off-clamp approach, dividing our objectives into two folds. Firstly, we compared trifecta outcomes of LPN and RAPN using the Hugo™ RAS System. 

The secondary objective was to assess the feasibility and safety of this innovative platform during the laparoscopic to robotic transition of a single surgeon with no prior robotic experience.

The concept of a “trifecta” for standardizing the outcomes of RAPN into a unified scoring system has become a useful tool for evaluating perioperative results and enhancing consistency across different studies. In recent times, various definitions and enhancements of the original trifecta have emerged; however, the diverse criteria used by different authors to assess acute kidney injury (AKI) may have significantly influenced reported outcomes. The absence of a standardized definition for key surgical factors makes it challenging to objectively measure trifecta achievement, leading to potential inaccuracies in result descriptions. However, the reproducibility of off-clamp procedures remains uncertain, and their predictive role in both oncologic and functional outcomes has not been directly compared or extensively explored in large cohorts. In this context, we incorporated a comprehensive trifecta for RAPN, which replaces warm ischemia time (WIT) with perioperative eGFR variations to accommodate clampless procedures [20].

Despite a higher conversion rate to an open approach being reported in the study by Choi J.E. et al. during laparoscopy, we did not experience any case requiring conversion [33]. 

The laparoscopic to robotic transition showed satisfactory and comparable outcomes between the two groups, similarly to the current literature. In fact, EBL was comparable between the two cohorts (*p* = 0.20), as were perioperative complications (*p* = 0.07), positive surgical margins rate (3.7% vs. 4.8%, *p* = 0.82), and eGFR at discharge and at the last follow-up (*p* = 0.85, 0.97; respectively). Group A also exhibited a shorter length of stay (3 vs. 5 days, *p* = 0.002), indicating potentially faster recovery. Operative time was favorable in group A (92 vs. 149.5 min, *p* = 0.005), configuring surgical and anesthesiologic advantages for patients. Moreover, trifecta was achieved in 92.6% of patients for group A and 82.3% for group B, while in 85.4% considering the entire cohort, without statistically significant differences (*p* = 0.10). 

In terms of pathology, we observed malignancies in 64.1% of the cases. Notably, among benign pathologies, oncocytoma was discovered in 22.5% of the patients. These findings align with the existing literature, particularly regarding tumors smaller than 4 cm, where the incidence increases to around 18% [6]. The diagnostic accuracy of imaging modalities such as CT scan and/or MRI in identifying renal oncocytoma is limited, with histopathology remaining the only reliable diagnostic modality.

To the best of our knowledge, this study represents the first comparative and comprehensive analysis of outcomes between LPN and RAPN performed by the Hugo™ RAS, providing valuable evidence to enhance the current literature. However, our study is not devoid of limitations. It was retrospective and conducted at a single center, with a limited sample size. Furthermore, the exclusion of data concerning assistant and department experience may have impacted perioperative parameters. Moreover, the homogeneity of the two cohorts may have minimized confounders arising from the small sample size. Notwithstanding these limitations, the analysis was focused on a single surgeon with extensive experience in minimally invasive surgery. While this may have ensured technique efficiency and reduced bias from varying surgical experiences, it is important to acknowledge the potential influence of individual skill levels on outcomes.

## 5. Conclusions

Partial nephrectomy is the preferred treatment for renal masses whenever technically feasible, with both laparoscopic and robotic approaches being reasonable options. The transition from LPN to RAPN for a trained urologist is not associated with any increase in positive surgical margins or complications; instead, it may be characterized by shorter operative times and length of stay. These findings may suggest that robotic surgery mimics natural movements and is likely to be quickly mastered.

Hugo™ RAS RAPNs showed comparable trifecta achievement outcomes in comparison to LPN when performed by the same experienced surgeon. The transition from LPN to RAPN using this novel robotic platform appears to be safe and uneventful, and these findings may potentially encourage naïve surgeons with previous laparoscopic experience to embrace robotic platforms.

## Figures and Tables

**Table 1:** Baseline and demographic for RAPN and Laparoscopic PN.

Variable	Overall (*n* = 89)	RAPN (*n* = 27) Group A	Laparoscopic PN (*n* = 62) Group B	*p*-Value
Age (*n*, median, IQR)	66 (55–72)	68 (57–73)	65.5 (54–72)	0.55
Gender (*n*, %)				
- Male - Female	- 59 (66.3%) - 30 (33.7%)	- 22 (81.5%) - 5 (18.5%)	- 37 (59.7%) - 25 (40.3%)	0.04
BMI (kg/m^2^, median, IQR)	27 (24.3–30.1)	27.4 (25.9–31.2)	26.3 (24–30.1)	0.19
ASA score (*n*, %)				
- I - II - III - IV	- 1 (1.1%) - 69 (77.6%) - 18 (20.2%) - 1 (1.1%)	- 1 (3.7%) - 17 (63%) - 9 (33.3%) - 0 (0%)	- 0 (0%) - 52 (83.9%) - 9 (14.5%) - 1 (1.6%)	0.06
Charlson Comorbidity Index (median, IQR)	4 (4–5)	4 (3–5)	4 (4–5)	0.81
Diabetes (*n*, %)	12 (13.5%)	2 (7.4%)	10 (16.1%)	0.26
Hypertension (*n*, %)	48 (53.9%)	14 (51.8%)	34 (54.8%)	0.79
Preoperative Hemoglobin (g/dL, median, IQR)	14.7 (13.3–15.4)	14.7 (12.3–15.4)	14.6 (13.2–15.4)	0.77
Preoperative Creatinine (mg/dL, median, IQR)	0.91 (0.76–1.02)	0.93 (0.81–1.09)	0.90 (0.73–1.00)	0.17
Preoperative eGFR (mL/min/1.73 m^2^, median, IQR)	84.6 (69.8–26.5)	77.5 (64.2–92.3)	85.6 (70.3–99.8)	0.08
Clinical Tumor Size (mm, median, IQR)	31 (20–44)	34 (26–45)	29 (19–44)	0.14
cT (*n*, %)				
- T1a - T1b - T2a - T2b - T3a	- 62 (69.7%) - 19 (21.3%) - 5 (5.6%) - 1 (1.1%) - 2 (2.3%)	- 21 (77.8%) - 3 (11.1%) - 2 (7.4%) - 2 (3.7%) - 0 (0%)	- 41 (66.2%) - 16 (25.8%) - 3 (4.8%) - 0 (0%) - 2 (3.2%)	0.22
Side (*n*, %)				
- Right - Left - Bilateral	- 51 (57.3%) - 35 (39.3%) - 3 (3.4%)	- 16 (59.3%) - 11 (40.7%) - 0 (0%)	- 35 (56.5%) - 24 (38.7%) - 3 (4.8%)	

				0.51
R.E.N.A.L. score (median, IQR)	6 (5–7)	7 (5–8)	6 (5–7)	0.11

**Table 2:** Perioperative data for RAPN and Laparoscopic PN.

Variable	Overall (*n* = 89)	RAPN (*n* = 27) Group A	Laparoscopic PN (*n* = 62) Group B	*p*-Value
Operative Time (min, median, IQR)	135 (75–197)	91 (50–149)	149.5 (83–203)	0.005
Estimated blood loss (mL, median, IQR)	100 (50–300)	150 (50–450)	100 (50–200)	0.20
Perioperative complications (*n*, %)	9 (10.1%)	3 (11.1%)	6 (9.7%)	0.07
Length of Stay (days, median, IQR)	4 (3–5)	3 (3–4)	5 (4–5)	0.002
Hemoglobin at discharge (g/dL, median, IQR)	11.6 (10.4–12.5)	11.2 (9.1–12.3)	11.8 (10.5–12.7)	0.09
Creatinine at discharge (mg/dL, median, IQR)	0.93 (0.77–1.1)	0.93 (0.82–1.13)	0.92 (0.75–1.1)	0.67
eGFR at discharge (mL/min/1.73 m^2^, median, IQR)	79.5 (63–92.1)	74.9 (63–92.1)	80.1 (63–92.1)	0.85
Readmission (*n*, %)	0 (0%)	0 (0%)	0 (0%)	-
Pathological Size (mm, median, IQR)	30 (14–40)	30 (20–43)	30 (13–40)	0.23
Pathology (*n*, %)				
- *Benign* - *Malignant*	- 32 (35.9%) - 57 (64.1%)	- 6 (22.2%) - 21 (77.8%)	- 26 (41.9%) - 36 (58.1%)	
				0.07
Histology_subtype (*n*, %)				
- Angiomyolipoma - Oncocytoma - *Clear Cell RCC* - *Papillary RCC* - *Cromophobe* - *Other*	- 8 (9%) - 20 (22.5%) - 37 (41.6%) - 10 (11.2%) - 9 (10.1%) - 5 (5.6%)	- 2 (7.4%) - 4 (14.8%) - 14 (51.9%) - 5 (18.5%) - 2 (7.4%) - 0 (0%)	- 6 (9.7%) - 16 (25.8%) - 23 (37.1%) - 5 (8.05%) - 7 (11.3%) - 5 (8.05%)	0.27
Positive Margins (*n*, %)	4 (4.5%)	1 (3.7%)	3 (4.8%)	0.82
pT Stage (*n*, %)				
- *1a* - *1b* - *2a* - *2b* - *3a*	- 59 (66.3%) - 17 (19.1%) - 3 (3.4%) - 1 (1.1%) - 9 (10.1%)	- 20 (74.1%) - 3 (11.1%) - 2 (7.4%) - 1 (3.7%) - 1 (3.7%)	- 39 (62.9%) - 14 (22.6%) - 1 (1.6%) - 0 (0%) - 8 (12.9%)	0.11
Last follow-up (months, median, IQR)	5 (1–9)	2 (1–5)	5 (1–9)	
Creatinine at last follow-up (mg/dL, median, IQR)	0.98 (0.85–1.13)	0.99 (0.85–1–14)	0.97 (0.79–1.08)	0.53
eGFR at last follow-up (mL/min/1.73 m^2^, median, IQR)	79.3 (58.7–88.7)	79.3 (58.3–87.8)	78 (60.6–90)	0.97
Trifecta achievement rate (%)	71 (85.4%)	25 (92.6%)	51 (82.3%)	0.10

## Data Availability

The data presented in this study are available on request from the corresponding author.
